# A novel, complex systems approach to modelling risk of psychological distress in young adolescents

**DOI:** 10.1038/s41598-021-88932-y

**Published:** 2021-05-03

**Authors:** Denise Beaudequin, Paul Schwenn, Larisa T. McLoughlin, Marcella Parker, Amanda Boyes, Gabrielle Simcock, Jim Lagopoulos, Daniel F. Hermens

**Affiliations:** Thompson Institute, University of the Sunshine Coast, Locked Bag 4 (ML59), Maroochydore DC, QLD 4558 Australia

**Keywords:** Psychology, Statistics

## Abstract

Adolescence is a period of significant anatomical and functional brain changes, and complex interactions occur between mental health risk factors. The Longitudinal Adolescent Brain Study commenced in 2018, to monitor environmental and psychosocial factors influencing mental health in 500 adolescents, for 5 years. Participants are recruited at age 12 from the community in Australia’s Sunshine Coast region. In this baseline, cross-sectional study of N = 64 participants, we draw on the network perspective, conceptualising mental disorders as causal systems of interacting entities, to propose a Bayesian network (BN) model of lifestyle and psychosocial variables influencing chances of individuals being psychologically well or experiencing psychological distress. Sensitivity analysis of network priors revealed that psychological distress (Kessler-10) was most affected by eating behaviour. Unhealthy eating increased the chance of moderate psychological distress by 600%. Low social connectedness increased the chance of severe psychological disorder by 200%. Certainty for psychological wellness required 33% decrease in unhealthy eating behaviours, 11% decrease in low social connectedness, and 9% reduction in less physical activity. BN can augment clinician judgement in mental disorders as probabilistic decision support systems. The full potential of BN methodology in a complex systems approach to psychopathology has yet to be realised.

## Introduction

Recognition of the potential of artificial intelligence (AI) techniques for clinical modelling and decision-making in medicine and psychiatry is increasing. Simultaneously, there is growing interest in understanding psychological disorders as complex systems, with emergence of the network approach to psychopathology^1,2^. Novel statistical methods reflecting the multifarious influences on, and dynamic nature of, psychopathology are needed to provide greater insight in complex mental health trajectories^3^. McGorry et al.^3^ highlight the need for new diagnostic and early identification strategies to overcome limitations of current analytic approaches, calling for innovative modelling and prediction strategies for mental health outcomes. Bayesian methods are increasingly employed in various statistical frameworks in psychology and related disciplines, including hypothesis testing, item response theory, and structural equation modelling^4^. Bayesian networks (BN) offer a systems approach to modelling risk and supporting decision making in complex domains, such as mental health trajectories^5,6^. Here we present a prototype BN for risk of psychological distress, based on self-reported lifestyle and psychosocial variables from a community-derived cohort of 12-year-old adolescents.

### Network perspective on psychopathology

The network approach to psychopathology is experiencing a surge of interest since its emergence in the last 10–15 years^7–9^, conceivably due to factors such as an exponential rise in data availability and moves in science and medicine away from reductionism and towards a systems perspective. The approach is based on the view that mental disorders arise as a result of complex interactions between psychological, biological and sociological elements, in conjunction with risk factors and symptoms. Patterns of interaction can be observed in a network structure, in which variables (e.g., symptoms, comorbidities, environmental or risk factors) are represented as nodes connected by arcs, implying the existence of a statistical association. Freese and Baer-Bositis^10^ describe psychopathology as networks of ‘problems’—adverse social, psychological, and genetic influences in individuals—connected by causal arcs, with the network approach facilitating focus on priority interventions or treatment targets to prevent propagation of problems. Isvoranu^11^ also suggests that personalised network modelling could be beneficial for intervention planning in psychotic disorders.

### Bayesian networks (BN)

BN are powerful risk assessment tools, particularly valuable for reasoning under uncertainty^5,12^, and are increasingly recognised as useful for prediction in complex systems such as environmental and health domains^13^. Importantly, Bayesian methods are able to produce reasonable results even with small to moderate sample sizes, particularly when robust prior information is available^14,15^.

A BN is a probabilistic graphical model representing a set of variables and their conditional dependencies via a directed acyclic graph (DAG). The variables, represented as nodes, are connected by directed arcs implying causality^5,6,12^. In a DAG, nodes with no parents are referred to as root nodes. If there is a directed arc from node Y to node Z, Y is said to be a *parent* of Z; likewise, Z is called a *child* of Y. Chance nodes in a BN have a number of user-defined ‘states’ that can be qualitative or discrete (e.g., ‘Yes/No, ‘High/Low’, ‘ > 5/ ≤ 5’). Conditional probabilities are assigned to each state, derived from data, simulation, or expert opinion, and algorithms compute the joint probability distribution of the network. Once quantified, BN can simulate multiple risk pathways or intervention scenarios, providing conditional probability of mental health outcomes. This interactive function of BN is achieved by changing the evidence ‘conditions’ in the network, wherein the BN is instantly updated. The many applications of BN models include comparing relative risks of scenarios, studying interactions between variables, quantifying the strength of associations, revealing obscure relationships and identifying sensitivities for target nodes.

BN are widely used in medicine for individual-level risk estimation and decision support^16^, and are increasingly employed in psychology, psychiatry and neuroscience^17–21^. BN offer a systems approach to decision making under uncertainty in complex domains^5,6^ boasting several advantages over traditional analytical techniques. In a comparative evaluation of frequentist and machine learning methods (BN) in a clinical trial of pravastatin, Cleophas and Zwinderman^22^ concluded that, compared with frequentist methods (t-tests, linear regressions), the machine learning methods provided better sensitivity of testing, were robust with respect to overfitting, efficient in describing multivariate distributions, and were more informative.

### The longitudinal adolescent brain study

The Longitudinal Adolescent Brain Study (LABS) commenced in July 2018 with the goal of monitoring changes in social, demographic, cognitive and psychological factors thought to influence or reflect mental health status in adolescents. Building on our precedent work^23^, the current paper examines interactions between risk and protective factors from participants’ self-report data, with the aim of better understanding the inter-relationships of self-reported states and influences on mental health. We present a prototype BN for risk of psychological distress for a community-derived sample of 12-year old adolescents, based on early findings from data in the first year of LABS. The BN explores relationships between risk factors able to be externally modulated (sleep, physical activity, social connectedness, eating behaviours), measures of internal homeostasis (impulsivity, metacognition, mindfulness), and an established measure of psychological distress (Kessler Psychological Distress Scale; K10), with the aim of exploring influences on risk of psychological distress in this sample.

## Method

### Study design and setting

The LABS enrols young people from the general population who are in their first year of high school (grade 7), from a range of public, private and independent schools in the local community. Young people and their caregivers are invited to contact LABS if they are interested in participating in the study. The study protocol was approved by the Human Research Ethics Committee, University of the Sunshine Coast (A181064). Informed consent was obtained from individual participants and their caregivers and the study was conducted in accordance with the Declaration of Helsinki. Participants were assessed at baseline and invited to return for assessments every four months for five years during the high school years. This paper focuses on data from the self-report questionnaire collected at baseline, during the first eight months of the study, at which point this paper was written. At this time in the study, 129 expressions of interest in participating had been received. Of these, 94 potential participants were screened, and 68 participants were enrolled in the study. At the time of this paper, *n* = 64 participants had completed their baseline assessment.

### Inclusion and exclusion criteria

Young people aged 12 years 0 months to 12 years 11 months, living in the Sunshine Coast region were included in the study. Young people with a major neurological disorder (e.g., epilepsy), intellectual disability, major medical illness, or who had sustained head injury with loss of consciousness > 30 min were excluded from the study.

### Data collection and preparation

The self-report questionnaire was administered to participants on a touch screen tablet, using the Qualtrics survey platform (Qualtrics, Provo, UT, USA 2019). The SPSS version 24.0 (IBM Corp 2016) was used for data preparation in conjunction with the Python programming package SciPy 1.4.1^24^.

### Variables used in the current study

The selection of variables for the current study was informed by our previous work^23^, examining associations between intrinsic and extrinsic variables thought to influence, or be influenced by, psychological distress. Variables from the LABS used in the current study are described in Table 1. The complete set of variables from the LABS self-report questionnaire is detailed elsewhere^23^.

**Table 1:** LABS variables used to model risk of psychological distress.

Construct	Measure	Notes	Interpretation
Quality of Life (QOL)	23-item World Health Organization Quality of Life (WHOQOL-BREF)	Four domains: (i) ‘physical health’, referring to energy and fatigue, pain and discomfort, and sleep and rest; (ii) ‘psychological’, referring to bodily image and appearance, negative feelings, positive feelings, self-esteem and thinking, learning, memory and concentration; (iii) ‘social relationships’, referring to personal relationships and social support; (iv) ‘environment’, referring to financial resources, freedom, physical safety and security, health and social care accessibility and quality, home environment, opportunities for acquiring new information and skills, and physical environment (pollution, noise, traffic, climate)^25^	In all domains, higher scores indicate higher quality of life
Physical Activity	3 items from Health Behaviour in School-Aged Children: WHO Collaborative Cross-national Study (HBSC)	^26^	Higher scores indicate higher levels of physical activity
Eating Behaviours	6 items from Food Frequency Questionnaire (FFQ); Health Behaviour in School-Aged Children: WHO Collaborative Cross-national Study (HBSC)	^26^	Higher scores indicate healthier eating habits
Sleep Quality	18-item Pittsburgh Sleep Quality Index (PSQI)	Assesses seven components of sleep quality: subjective sleep quality, sleep duration, sleep latency, habitual sleep efficiency, sleep disturbance, use of sleep medication, and daytime dysfunction	A higher total score indicates higher sleep dysfunction, with scores ≤ 5 indicating good sleep patterns and quality
Metacognition	30-item Metacognition Questionnaire-Adolescent version (MCQ-A)	Assesses thought processes involved in monitoring one’s thinking, specifically intrusive thinking and worry^27^	Higher total scores reflect a stronger presence of beliefs about metacognitive processes
Psychological Distress	10-item Kessler Psychological Distress Scale (K10)	Widely used screening tool in primary care for symptoms of depression and anxiety over the past 4 weeks^28^	Total scores range from 10 – 50 and established cut-offs of the summed scores indicate the likelihood of the participant being psychologically well (scores < 20); or experiencing mental disorder of a ‘mild’ (scores 21 – 24), ‘moderate’ (scores 25–29), or ‘severe’ (scores ≥ 30) level
Social Connectedness	15-item Social Connectedness Scale (SCS)	Assesses participants’ ability to feel comfortable, confident, and have a sense of belonging within a larger social context than family or friends	Higher scores indicate that the participant feels more socially connected
Cyberstrife	Derived from the 35-item Berlin Cyberbullying-Cybervictimization Questionnaire (BCCQ)	A single, dichotomous category ‘Cyberstrife’ was derived from BCCQ data, based on whether participants reported being a cyberbully, cybervictim or both bully and victim	Cyberbully, cybervictim or both; Not cyberbully, cybervictim or both
Mindfulness	14-item Mindful Attention Awareness Scale (MAAS-A)	Measures awareness of and attention to what is taking place in the present^29^	Higher scores indicate higher (healthier) mindfulness traits
Impulsivity	8-item Barratt Impulsiveness Scale—Brief (BIS-Brief)	Provides a unidimensional measure of general impulsiveness^30^	Higher scores indicate higher (unhealthier) impulsivity

### Model structure and parameterisation

A conceptual model was created using GeNIe 2.3.3828.0 (BayesFusion, LLC; http://www.bayesfusion.com/). Selection of variables for the conceptual model in the current study was informed initially by our previous work^23^, examining associations between measures of intrinsic homeostasis and extrinsic modulators thought to influence (or be influenced by) psychological distress. The structure of the conceptual model was developed iteratively from the complete suite of LABS self-report variables, guided by the domain knowledge of the LABS team, with the primary aim of studying the interacting effects of self-reported risk and protective factors on chance of psychological distress. Parsimony, a primary goal in BN design, where the simplest structure to describe the system being studied is the best^31^, was a guiding principle in model development.

The primary outcome measure chosen was psychological distress, measured by the K10, with other self-report variables used as inputs to the model. Dichotomous states were chosen for all variables except the outcome variable, K10, where established categories were used^28,32^. Threshold ranges for node states (Table S1, supplementary file) were selected where possible from validated ranges in the literature. Eleven measures without validated ranges were discretised using percentiles of the possible range for each measure (< 50th, and ≥ 50th for low and high categories, respectively). The states of parentless or root nodes were parameterised using frequency distributions from the LABS data. The conditional probabilities underlying child nodes in the BN were derived using Bayes' theorem, which evaluates the probability of an event, based on conditions that are thought to influence the event. Mathematically, Bayes’ theorem is represented by:

1 $$ \begin{array}{*{20}c} {P\left( {B{|}A} \right) = \frac{{P\left( {A{|}B} \right)P\left( B \right)}}{P\left( A \right)} } \\ \end{array} $$

where $P\left( {B|A} \right)$ is the conditional probability of event $B$, given that event $A$ is true. Parameterisation of the states of child nodes was accomplished by calculating conditional probabilities from the data set. The BN model structure, and discretisation and parameterisation of nodes was reviewed and modified by domain experts from the disciplines of psychology and neurobiology and validated by mental health clinicians.

### Model validation

The BN was evaluated through inference, scenario analysis and sensitivity analysis. Validation of model behaviour was accomplished by cross-checking the probabilities in the network with each other for consistency, such that if the probabilities in one node of the network were changed, the subsequent changes in probabilities of other nodes followed approximately as expected. Sensitivity analysis, described in the next section, completed the model validation procedures, in the absence of other Bayesian networks in this domain with which to compare model output^33^.

### Baseline network and sensitivity assessment

**Figure 1:**
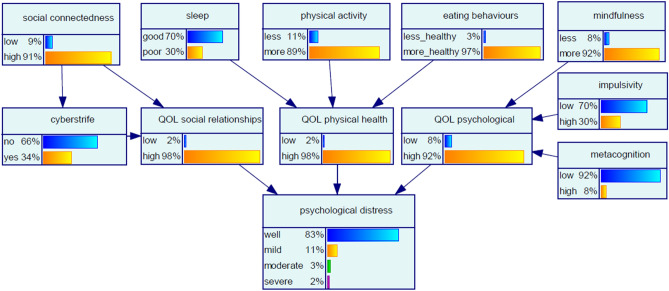
Baseline Bayesian network for risk of psychological distress.

The network in Fig. 1 presents the prior probability distribution of each node, computed from the relationships of influence and resulting conditional probabilities. Figure 1 thus represents the reference point for the network, i.e., before any further evidence is added. The value ascribed to a node state represents the chance that the node will be in that state. For example, in the study population at the baseline assessment, the chance of a participant being psychologically well as indicated by the *psychological distress* node is 83%, and as indicated in the *physical activity* node, the chance that a participant is less active is 11%. Sensitivity analysis of the model priors was conducted using an algorithm proposed by Kjaerulff and van der Gaag^34^ to give an indication of the relative importance of model inputs in terms of precision. The assessment showed that, in the absence of introduced evidence, the target node *psychological distress* was most sensitive to the modifiable node *eating behaviours*, followed by *social connectedness,* indicating that small changes in these nodes may lead to a large change in the posterior of the target node.

### Scenarios

An established practice in BN modelling is consideration of various scenarios (or queries), by setting evidence in input nodes, in order to draw conclusions about another node under those conditions. Evidence is introduced in a single node by selecting a node state. To complete model verification, the probability distribution in the outcome node is observed in response to varying combinations of parameters in input nodes. Multivariate scenarios are presented in the Results, demonstrating modelling of multiple influences on risk of psychological distress.

### Statistical analysis

Categorical variables were described in terms of frequencies and percentages; continuous variables were summarised as means ± standard deviation (SD). To study the effect of new evidence introduced into the network on the chosen outcome measures, the percent change (${\Delta }_{\%}$) in each response node state was calculated as

2 $$ {{\Delta }_{\% } = \frac{{P_{\text{evidence}}- P_{\text{baseline}}}}{{P_{{{\text{baseline}}}} }} \times 100\% }
$$

where $P_{{{\text{baseline}}}}$ is the probability of occurrence of response node states under baseline network conditions before new evidence is introduced and $P_{{{\text{evidence}}}}$ is the probability of a state occurring after new evidence is introduced into the network^35^.

## Results

### Sample description

64 participants aged 12 years and in grade 7 were recruited between July 2018 and February 2019. Forty seven percent (*n* = 30) of participants were female. The mean (SD) height for the sample was 157 (6.8) cm, the mean (SD) weight was 47 (9.5) kg and mean (SD) BMI was 19 (3.0). Fifty two percent of participants (*n* = 33) attended government schools, 20% of participants (*n* = 13) attended independent schools, 27% of participants (*n* = 17) attended religious schools and 1 participant attended distance education. Thirty one percent of participants (*n* = 20) had consulted a mental health professional at least once in their lifetime. Of these, seven participants had formal diagnoses for a developmental disorder—four participants had a diagnosis of autism spectrum disorder and three participants had been diagnosed as having ADHD. Four participants were taking psychopharmacological medication. Summary data (*n* = 64) from LABS used in the BN are presented in Table 2.

**Table 2:** LABS summary data used in BN for risk of psychological distress.

Variable	n	%
**Social connectedness**		
Low	6	9
High	58	91
**Sleep**		
Good	45	70
Poor	19	30
**Physical activity**		
Less	7	11
More	57	89
**Eating behaviours**		
Less healthy	2	3
More healthy	62	97
**Mindfulness**		
Less	5	8
More	59	92
**Cyberstrife**		
No	42	66
Yes	22	34
**QOL social relationships**		
Low	1	2
High	63	98
**QOL physical health**		
Low	1	2
High	63	98
**QOL psychological**		
Low	6	9
High	58	91
**Impulsivity**		
Low	45	70
High	19	30
**Metacognition**		
Low	59	92
High	5	8
**Psychological distress**		
Well	54	84
Mild	7	11
Moderate	2	3
Severe	1	2

### Scenario analyses

#### Scenario 1 ‘certainty for psychological wellness’

**Figure 2:**
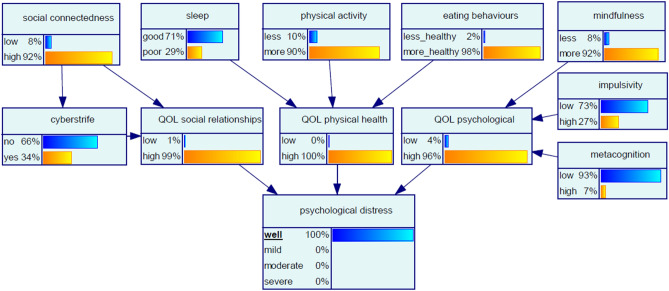
Bayesian network for Scenario 1 ‘certainty for psychological wellness’.

In the first instance, it is of interest to determine the conditions for certainty that a participant will be psychologically well, simulated by setting the *psychological distress* node to 100% ‘well’. To ensure the query constraint, multiple parameters in the network changed simultaneously (Fig. 2). Table 3 summarises the percent change in each input node in response to the query constraint. The variables amenable to external modulation with the largest changes required to achieve the target of 100% certainty for a participant to be well were eating behaviours, social connectedness and physical activity. Risk factors such as cyberstrife and sleep were not as influential in achieving psychological wellness in this sample. This scenario, and the next, ‘certainty for severe psychological distress’, are examples of the ‘backwards reasoning’ ability of BN, whereby the required state in the outcome node *psychological distress* is specified, and the states of the network required to obtain the required outcome are determined using priors and Bayes' theorem.

**Table 3:** Scenario 1 ‘certainty for psychological wellness’, displaying changes in Bayesian network node states to achieve certainty of a participant being psychologically well.

Node	${\text{Change }}\Delta_{\% }$*
**Social connectedness**	
Low	− 11
High	1
**Sleep**	
Good	1
Poor	− 3
**Physical activity**	
Less	− 9
More	1
**Eating behaviours**	
Less healthy	− 33
More healthy	1
**Mindfulness**	
Less	0
More	0
**Cyberstrife**	
No	0
Yes	0
**QOL social relationships**	
Low	− 50
High	1
**QOL physical health**	
Low	− 100
High	2
**QOL psychological**	
Low	− 50
High	4
**Impulsivity**	
Low	4
High	− 10
**Metacognition**	
Low	1
High	− 13

*$$\Delta _{\% } = \frac{{P_{{{\text{evidence }} - { }}} P_{{{\text{baseline}}}} }}{{P_{{{\text{baseline}}}} }} \times 100\%.$$

#### Scenario 2 ‘certainty for severe psychological distress’

**Figure 3:**
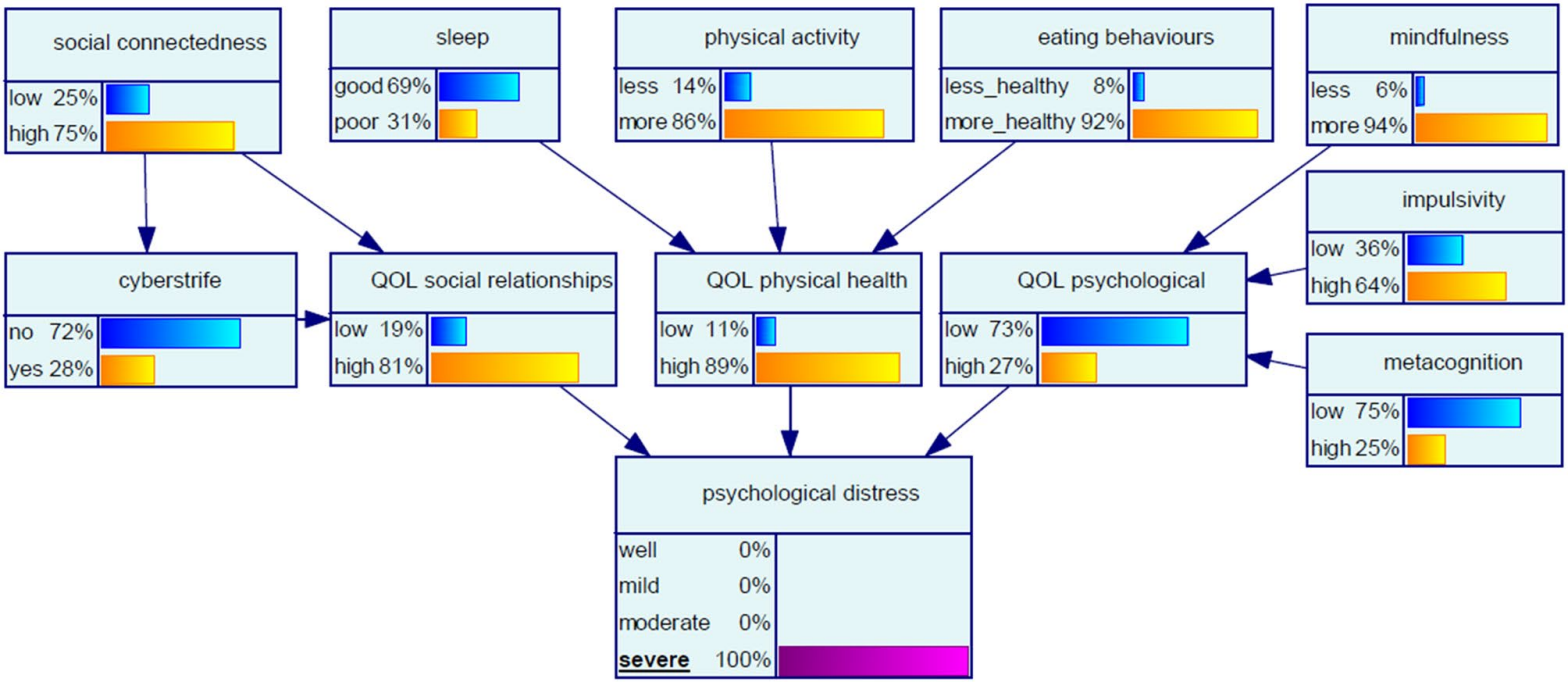
Bayesian network for Scenario 2 ‘certainty for severe psychological distress’.

We next explored network conditions for certainty of a participant having severe psychological distress, modelled by setting the *psychological distress* node to ‘100% severe’ (Fig. 3). The changes in states observed in ‘upstream’ nodes in response to this evidence are shown in Table 4. The modifiable variable with the largest influence in this scenario was *social connectedness*. With certainty of severe psychological distress, the chance of low *social connectedness* increased by 178%. At the same time the chance of the participant experiencing *cyberstrife* decreased by 18%, feasibly due to the influence of decreased *social connectedness.* These findings are reflected in the *QOL social relationships* node, where the chance of low quality of life regarding social relationships increased by 850%. Also of note in this scenario, high levels of *impulsivity* and *metacognition* increased by 113% and 213%, respectively.

**Table 4:** Scenario 2 ‘certainty for severe psychological distress’, displaying changes propagated in Bayesian network with query constraint of severe psychological distress.

Node	${\text{Change}} \Delta_{\% }$*
**Social connectedness**	
Low	178
High	− 18
**Sleep**	
Good	− 1
Poor	3
**Physical activity**	
Less	27
More	− 3
**Eating behaviours**	
Less healthy	167
More healthy	− 5
**Mindfulness**	
Less	− 25
More	2
**Cyberstrife**	
No	9
Yes	− 18
**QOL social relationships**	
Low	850
High	− 17
**QOL physical health**	
Low	450
High	− 9
**QOL psychological**	
Low	813
High	− 71
**Impulsivity**	
Low	− 49
High	113
**Metacognition**	
Low	− 18
High	213

*$$\Delta _{\% } = \frac{{P_{{{\text{evidence }} - { }}} P_{{{\text{baseline}}}} }}{{P_{{{\text{baseline}}}} }} \times 100\%.$$

#### Scenario 3 ‘certainty for less healthy eating behaviours’

**Figure 4:**
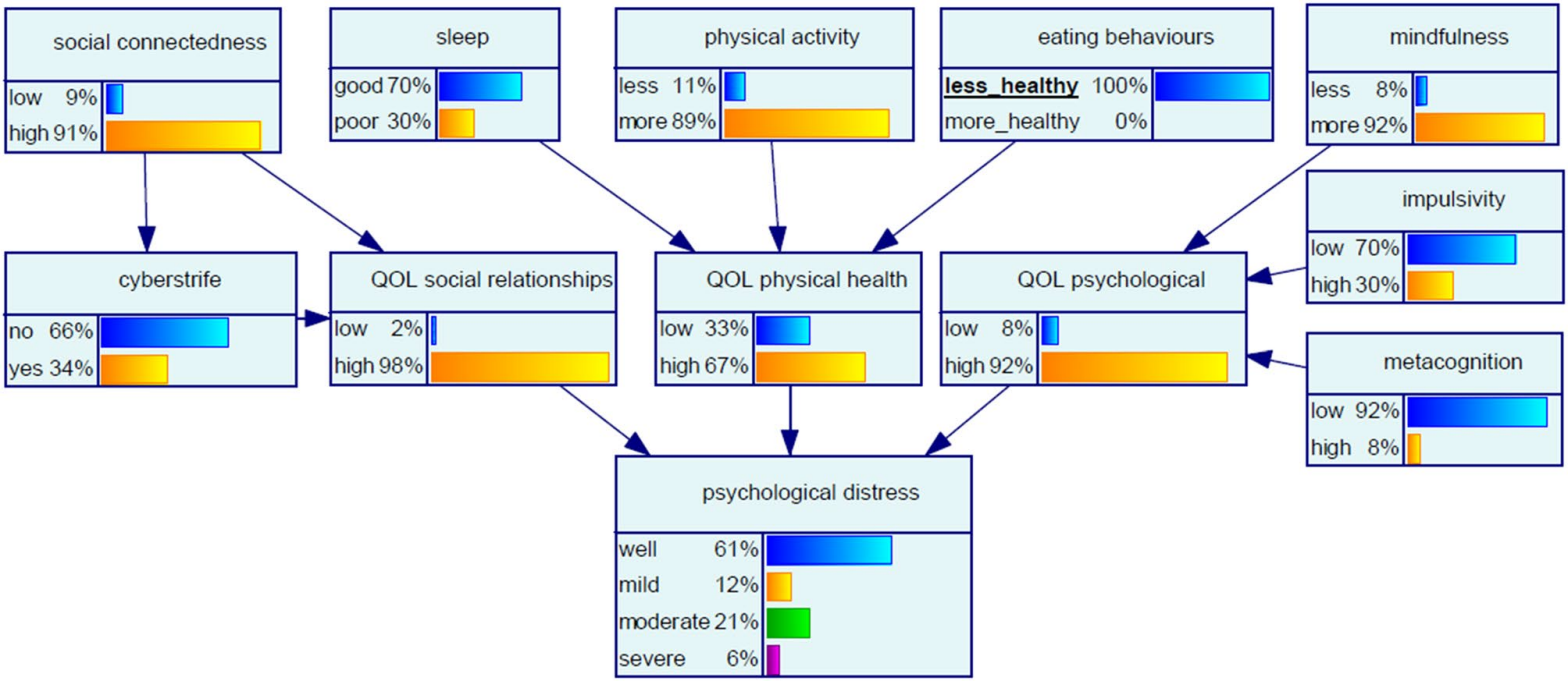
Bayesian network for Scenario 3 ‘certainty for less healthy eating behaviours’.

Increasing the chance of a participant adopting less healthy eating behaviours to certainty (100%) had a marked effect on the chance of a participant being psychologically well, which decreased by 27% (Fig. 4). The chance of a participant having psychological distress of moderate severity increased more significantly, by 600%. Certainty for less healthy eating behaviours also had a significant effect on the chance of a participant having low quality of life with respect to physical health, which increased from baseline by 1550%. The changes in affected nodes are shown in Table 5.

**Table 5:** Changes from baseline in affected nodes with Scenario 3 ‘certainty for less healthy eating behaviours’.

Node	State	Baseline %	Scenario ‘certainty for less healthy eating behaviours’ %	${\text{Change}} \Delta_{\% }$*
QOL physical health	Low	2	33	1550
	High	98	67	− 32
Psychological distress	Well	83	61	− 27
	Mild	11	12	9
	Moderate	3	21	600
	Severe	2	6	200

*$$\Delta _{\% } = \frac{{P_{{{\text{evidence }} - { }}} P_{{{\text{baseline}}}} }}{{P_{{{\text{baseline}}}} }} \times 100\%.$$

#### Scenario 4 ‘certainty for low social connectedness’

Setting the s*ocial connectedness* node to ‘100% low’, indicating certainty for low social connectedness had the ‘downstream’ effect of increasing the chance of cyberstrife by 26%, increasing the chance of low quality of life with respect to social relationships by 850% and reducing the chance of a participant being psychologically well by 11%. Notably, certainty for low social connectedness also increased the chance of severe psychological disorder by 200%. The changes in affected nodes are shown in Table 6.

**Table 6:** Changes from baseline in affected nodes with Scenario 4 ‘certainty for low social connectedness’.

Node	State	Baseline %	Scenario ‘certainty for low social connectedness’ %	${\text{Change}} \Delta_{\% }$*
QOL social relationships	Low	2	19	850
	High	98	81	− 17
Cyberstrife	No	66	57	− 14
	Yes	34	43	26
Psychological distress	Well	83	74	− 11
	Mild	11	13	18
	Moderate	3	7	133
	Severe	2	6	200

*$$\Delta _{\% } = \frac{{P_{{{\text{evidence }} - { }}} P_{{{\text{baseline}}}} }}{{P_{{{\text{baseline}}}} }} \times 100\%.$$

## Discussion

Using Bayesian analyses, this study sought to explicate the complex interactions between influences on mental health. Important findings that emerged were the conditions necessary for a participant to be certain of being psychologically well (Scenario 1). These conditions included a decrease in the chance of unhealthy eating behaviours by 33%; reduction in low social connectedness of 11%; and a decrease in the chance of less physical activity by 9%. Conversely, network conditions under which a participant was certain to be experiencing severe psychological distress (Scenario 2) included a 178% increase in the chance of low social connectedness, albeit accompanied by a decrease in the chance of cyberstrife by 18%.

The interactions between the *social connectedness* and *cyberstrif*e nodes were particularly interesting. Certainty for low social connectedness (Scenario 4) alone resulted in an increased chance of cyberstrife. This result is consistent with the finding by McLoughlin et al.^36^, that cyberbully-victims experienced lower levels of social connectedness than those who had never been involved in cyberbullying as victim or bully. Nonetheless, the network conditions required for the query constraint in Scenario 3 (severe psychological distress), included an increased chance of low social connectedness, and *decreased* chance of cyberstrife. This situation, wherein evidence entered in a node varies in its effect depending upon network conditions, is illustrative of the ability of a BN to model the mutual distribution of all states in all nodes in the network, considering node dependencies and any new evidence introduced to the network simultaneously. Thus, regardless of model response to evidence introduction in a single node, the same evidence may prompt a different response in a multivariate scenario, contingent on the combination of evidence in other nodes. Notably, our study findings highlighted the substantial effect of eating behaviours on psychological distress as measured by the K10. Modelling a participant adopting less healthy eating behaviours by setting the ‘less healthy’ *eating behaviours* node state to certainty had the effect of decreasing the chance of the participant being psychologically well by 27%. This result corroborates other evidence supporting links between food and mood in adolescents, from Australia^37–39^ and elsewhere^40–42^.

Examination of the LABS data on eating behaviour showed that 38% (*n* = 24) of participants did not eat fruit every day; 52% of participants (*n* = 33) ate vegetables more than once a day, every day; 73% (*n* = 47) of participants ate sweets (candy or chocolate) more than once per week; 14% (*n* = 9) of participants had soft drinks containing sugar more than once a week; 35% (*n* = 22) of participants didn’t have breakfast on at least one weekday, and 25% (*n* = 16) skipped breakfast on at least one weekend day. These insights, in conjunction with the probabilistic modelling of the interaction between eating behaviour and psychological distress evident in our community-derived sample of 12-year-olds, are indicative of strong potential for risk reduction strategies.

As we have shown, modelling of psychosocial and lifestyle data using BN provides rich insights into networks of risk and protective factors for psychopathology in young adolescents. BN can be built and estimated in several ways and used to examine simultaneous changes in variables and relative risks during scenario comparisons. A BN can reveal obscure relationships between variables and generate sensitivity profiles for different target nodes. BN probabilities are updated immediately when evidence is entered and the interactive, visual platform presents scenario outcomes plainly to users, regardless of their discipline. The ability of BN to present and evaluate multivariate scenarios is particularly beneficial in analysing complex systems. Hence BN, when used as probabilistic decision support systems, can complement clinician judgement in mental disorders with prediction and weighing treatment options^43,44^. The full potential of the BN methodology in a complex systems approach to psychopathology, with benefits for multidisciplinary teams, has yet to be realised.

Limitations in the current study suggest potential future directions for BN modelling in psychopathology research. Firstly, the network developed for the purposes of this study was constructed using variables derived from a self-report questionnaire administered to a self-selected sample of 12-year-old adolescents; our inferences about model behaviour must therefore be qualified accordingly. Secondly, the current study employs data from a single timepoint; incorporation of longitudinal data would enable assessment of the temporal role on risk and protective factors, symptoms and neurobiology. Thirdly, the BN presented here is a simple prototype, developed to demonstrate the potential of the approach in this domain. Further research could include inputs from other dimensions, such as indicators of functional impairment, neuroimaging biomarkers, measures of cognition, clinical inputs such as formal diagnoses, lifestyle factors such as screen time, and demographic variables such as family history of mental illness. Input nodes such as eating behaviours which are shown to have large impacts on target nodes can be modelled in more detail, with inclusion as sub models.

Lastly, small sample size is a limitation in the current study. However, LABS is a longitudinal study, aiming to recruit 500 participants in total. The BN presented here should be replicated, with a larger sample size. A larger sample size will also enable examination of gender differences.

The BN modelling in this study yielded both novel and previously validated findings regarding influences and risk factors for risk of psychological distress in adolescents. BN are sophisticated tools which can optimise use of multidimensional data in our understanding of psychopathology, transcending reductionism and biomedical approaches, and producing new perspectives and actionable insights in an indisputably complex field.

## Supplementary information

Supplementary Information.

## References

[1] Borsboom D (2017). A network theory of mental disorders. World Psychiatry.

[2] Borsboom D, Cramer AOJ, Kalis A (2019). Brain disorders? Not really: Why network structures block reductionism in psychopathology research. Behav. Brain Sci..

[3] McGorry PD, Hartmann JA, Spooner R, Nelson B (2018). Beyond the “at risk mental state” concept: transitioning to transdiagnostic psychiatry. World Psychiatry.

[4] van de Schoot R, Winter SD, Ryan O, Zondervan-Zwijnenburg M, Depaoli S (2017). A systematic review of Bayesian articles in psychology: the last 25 years. Psychol. Methods.

[5] Jensen FV, Nielsen TD (2007). Bayesian Networks and Decision Graphs.

[6] Pearl J (2000). Causality.

[7] Cramer A, Waldorp L, van der Maas H, Borsboom D (2010). Comorbidity: a network perspective. Behav. Brain Sci..

[8] Borsboom D (2008). Psychometric perspectives on diagnostic systems. J. Clin. Psychol..

[9] Fried EI (2017). Mental disorders as networks of problems: a review of recent insights. Soc. Psychiat. Psychiat. Epidemiol..

[10] Freese J, Baer-Bositis L (2019). Networks of problems: social, psychological, and genetic influences on health. Curr. Opin. Psychol..

[11] Isvoranu AM (2018). Symptom network models of psychosis. Schizophr. Bull..

[12] Pearl J (1988). Probabilistic Reasoning in Intelligent Systems: Networks of Plausible Inference.

[13] Lucas PJ, Gaag LC, Abu-Hanna A (2004). Bayesian Networks in biomedicine and health-care. Artif. Intell. Med..

[14] Zhang Z, Hamagami F, Wang L, Nesselroade JR, Grimm KJ (2007). Bayesian analysis of longitudinal data using growth curve models. Int. J. Behav. Dev..

[15] Van De Schoot R, Broere JJ, Perryck KH, Zondervan-Zwijnenburg M, Van Loey NE (2015). Analyzing small data sets using Bayesian estimation: the case of posttraumatic stress symptoms following mechanical ventilation in burn survivors. Eur. J. Psychotraumatol..

[16] Arora P (2019). Bayesian networks for risk prediction using real-world data: a tool for precision medicine. Value Health.

[17] Bilek G, Karaman F (2018). An investigation into the relationship among psychiatric, demographic and socio-economic variables with Bayesian network modeling. Entropy.

[18] Kuang D (2017). Depression recognition according to heart rate variability using Bayesian Networks. J. Psychiatr. Res..

[19] McNally RJ, Mair P, Mugno BL, Riemann BC (2017). Co-morbid obsessive-compulsive disorder and depression: a Bayesian network approach. Psychol. Med..

[20] Rodgers RF (2019). Structural differences in eating disorder psychopathology after history of childhood abuse: insights from a bayesian network analysis. J. Abnorm. Psychol..

[21] Ojeme B, Mbogho A (2016). Smart innovation. Syst. Technol..

[22] Cleophas TJ, Zwinderman AH (2019). Efficacy Analysis in Clinical Trials an Update: Efficacy Analysis in an Era of Machine Learning 75–85.

[23] Beaudequin D (2020). Using measures of intrinsic homeostasis and extrinsic modulation to evaluate mental health in adolescents: Preliminary results from the Longitudinal Adolescent Brain Study (LABS). Psychiat. Res..

[24] Virtanen P (2020). SciPy 1.0: fundamental algorithms for scientific computing in Python. Nat. Methods.

[25] World Health Organization. *WHOQOL: Measuring Quality of Life*, https://www.who.int/healthinfo/survey/whoqol-qualityoflife/en/index4.html (2019).

[26] Currie, C. *et al.* Health behaviour in school-aged children (HBSC) study protocol: background, methodology and mandatory items for the 2013/14 survey., (University of St Andrews: Child and Adolescent Health Research Unit 2014).

[27] Cartwright-Hatton S (2004). Development and preliminary validation of the meta-cognitions questionnaire: adolescent version. J. Anxiety Disord..

[28] Kessler RC (2002). Short screening scales to monitor population prevalences and trends in non-specific psychological distress. Psychol. Med..

[29] Brown KW, West AM, Loverich TM, Biegel GM (2011). Assessing adolescent mindfulness: validation of an adapted mindful attention awareness scale in adolescent normative and psychiatric populations. Psychol. Assess..

[30] Steinberg L, Sharp C, Stanford MS, Tharp AT (2013). New tricks for an old measure: the development of the Barratt Impulsiveness Scale-Brief (BIS-Brief). Psychol. Assess..

[31] Pollino C, Henderson C (2010). Bayesian Networks: A Guide for Their Application in Natural Resource Management and Policy.

[32] Andrews G, Slade T (2001). Interpreting scores on the Kessler psychological distress scale (K10). Aust. N. Z. J. Public Health.

[33] Beaudequin D, Harden F, Roiko A, Mengersen K (2016). Utility of Bayesian networks in QMRA-based evaluation of risk reduction options for recycled water. Sci. Total Environ..

[34] Kjaerulff, U. & van der Gaag, L. C. in *Sixteenth Conference on Uncertainty in Artificial Intelligence 2000.*

[35] Beaudequin D, Harden F, Roiko A, Mengersen K (2017). Potential of Bayesian networks for adaptive management in water recycling. Environ. Model. Softw..

[36] McLoughlin LT, Spears BA, Taddeo CM, Hermens DF (2019). Remaining connected in the face of cyberbullying: Why social connectedness is important for mental health. Psychol. Sch..

[37] Jacka FN (2010). Associations between diet quality and depressed mood in adolescents: results from the Australian healthy neighbourhoods study. Aust. N. Z. J. Psychiatry.

[38] Jacka FN (2011). A prospective study of diet quality and mental health in adolescents. PLoS ONE.

[39] Oddy WH (2009). The association between dietary patterns and mental health in early adolescence. Prev. Med..

[40] Liu MW (2020). Fruit and vegetable intake in relation to depressive and anxiety symptoms among adolescents in 25 low- and middle-income countries. J. Affect. Disord..

[41] Hoare E (2019). Lifestyle behavioural risk factors and emotional functioning among schoolchildren: the Healthy Growth Study. Eur. Psychiat..

[42] Oellingrath IM, Svendsen MV, Hestetun I (2014). Eating patterns and mental health problems in early adolescence–a cross-sectional study of 12–13-year-old Norwegian schoolchildren. Public Health Nutr..

[43] McNally, R. J. Mental Disorders as Causal Systems: A Network Approach to Posttraumatic Stress Disorder. (2015).

[44] Wichers M, Schreuder MJ, Goekoop R, Groen RN (2019). Can we predict the direction of sudden shifts in symptoms? Transdiagnostic implications from a complex systems perspective on psychopathology. Psychol. Med..

