# Supplementary material for: A novel, complex systems approach to modelling risk of psychological distress in young adolescents
Source: Sci Rep. 2021 May 3;11:9428. doi: 10.1038/s41598-021-88932-y (PMC8093239; doi:10.1038/s41598-021-88932-y)
Supplement: Supplementary file 1 — Supplementary Information. [file 41598_2021_88932_MOESM1_ESM.docx]

**A novel, complex systems approach to modelling risk of psychological distress in young adolescents**

*Denise Beaudequin^1^; Paul Schwenn^1^; Larisa T. McLoughlin^1^; Marcella Parker; Amanda Boyes^1^; Gabrielle Simcock^1^; Jim Lagopoulos^1^; Daniel F. Hermens^1^

^1^Thompson Institute, University of the Sunshine Coast, Locked Bag 4 (ML59), Maroochydore DC, QLD 4558, Australia

Corresponding author: Denise Beaudequin, Thompson Institute, University of the Sunshine Coast, Locked Bag 4 (ML59), Maroochydore DC, QLD 4558, Australia, dbeaudeq@usc.edu.au, +61 7 5459 4876

**Supplementary file**

Table S1. Thresholds for node states – Bayesian network for risk of psychological distress, Longitudinal Adolescent Brain Study (LABS) baseline assessment (n = 64)

| **domain** | **measure** | **possible min - max** | **thresholds for states** |
| --- | --- | --- | --- |
| social connectedness | SCS | 15 - 90 | low = 15 - 52  high = 53 - 90 |
| sleep | PSQI Total | 0 - 21 | good = 0 - 5  poor = 6 - 21 |
| physical activity | HBSC | 0 - 18 | less = 0 – 8  more = 9 - 18 |
| eating behaviours | FFQ | 0 - 31 | less healthy = 0 - 15  more healthy = 16 - 31 |
| mindfulness | MAAS-A | 14 - 84 | less = 14 – 48  more = 49 - 84 |
| cyberstrife | BCCQ | cyberbullying  18 – 90  cybervictimisation 17 - 85 | no = not a bully, a victim or a bully-victim  yes = a bully, a victim or a bully-victim |
| quality of life | WHOQOL-BREF social relationships | 2 - 10 | low = 2 - 5  high = 6 - 10 |
|  | WHOQOL-BREF physical health | 5 - 25 | low = 5 - 14  high = 15 - 25 |
|  | WHOQOL-BREF psychological | 6 - 30 | low = 6 - 17  high = 18 - 30 |
| impulsivity | BIS-Brief | 8 - 32 | low = 8 - 19  high = 20 - 32 |
| metacognition | MCQ-A Total | 30 - 120 | low = 30 – 74  high = 75 - 120 |
| psychological distress | K10 | 10 - 50 | well = < 20  mild = 20 - 24  moderate = 25 - 29  severe = ≥ 30 |
